# Machine Learning and Radiomics of Bone Scintigraphy: Their Role in Predicting Recurrence of Localized or Locally Advanced Prostate Cancer

**DOI:** 10.3390/diagnostics13213380

**Published:** 2023-11-03

**Authors:** Yu-De Wang, Chi-Ping Huang, You-Rong Yang, Hsi-Chin Wu, Yu-Ju Hsu, Yi-Chun Yeh, Pei-Chun Yeh, Kuo-Chen Wu, Chia-Hung Kao

**Affiliations:** 1 Graduate Institute of Biomedical Sciences, School of Medicine, College of Medicine, China Medical University, Taichung 404327, Taiwan; 027065@tool.caaumed.org.tw; 2 Department of Urology, China Medical University Hospital, Taichung 404327, Taiwan; 017561@tool.caaumed.org.tw (C.-P.H.); u9701063@gmail.com (Y.-R.Y.); 3 School of Medicine, China Medical University, Taichung 406040, Taiwan; 004746@tool.caaumed.org.tw; 4 Department of Urology, China Medical University Beigang Hospital, Yunlin 651012, Taiwan; 5 Artificial Intelligence Center, China Medical University Hospital, Taichung 404327, Taiwan; 035456@tool.caaumed.org.tw (Y.-J.H.); 024118@tool.caaumed.org.tw (Y.-C.Y.); 008252@tool.caaumed.org.tw (P.-C.Y.); 029699@tool.caaumed.org.tw (K.-C.W.); 6 Graduate Institute of Biomedical Electronics and Bioinformatics, National Taiwan University, Taipei 106319, Taiwan; 7 Department of Nuclear Medicine and PET Center, China Medical University Hospital, Taichung 404327, Taiwan; 8 Department of Bioinformatics and Medical Engineering, Asia University, Taichung 413305, Taiwan

**Keywords:** machine learning, bone scintigraphy, radiomics, prostate cancer, recurrence

## Abstract

Background: Machine-learning (ML) and radiomics features have been utilized for survival outcome analysis in various cancers. This study aims to investigate the application of ML based on patients’ clinical features and radiomics features derived from bone scintigraphy (BS) and to evaluate recurrence-free survival in local or locally advanced prostate cancer (PCa) patients after the initial treatment. Methods: A total of 354 patients who met the eligibility criteria were analyzed and used to train the model. Clinical information and radiomics features of BS were obtained. Survival-related clinical features and radiomics features were included in the ML model training. Using the pyradiomics software, 128 radiomics features from each BS image’s region of interest, validated by experts, were extracted. Four textural matrices were also calculated: GLCM, NGLDM, GLRLM, and GLSZM. Five training models (Logistic Regression, Naive Bayes, Random Forest, Support Vector Classification, and XGBoost) were applied using K-fold cross-validation. Recurrence was defined as either a rise in PSA levels, radiographic progression, or death. To assess the classifier’s effectiveness, the ROC curve area and confusion matrix were employed. Results: Of the 354 patients, 101 patients were categorized into the recurrence group with more advanced disease status compared to the non-recurrence group. Key clinical features including tumor stage, radical prostatectomy, initial PSA, Gleason Score primary pattern, and radiotherapy were used for model training. Random Forest (RF) was the best-performing model, with a sensitivity of 0.81, specificity of 0.87, and accuracy of 0.85. The ROC curve analysis showed that predictions from RF outperformed predictions from other ML models with a final AUC of 0.94 and a *p*-value of <0.001. The other models had accuracy ranges from 0.52 to 0.78 and AUC ranges from 0.67 to 0.84. Conclusions: The study showed that ML based on clinical features and radiomics features of BS improves the prediction of PCa recurrence after initial treatment. These findings highlight the added value of ML techniques for risk classification in PCa based on clinical features and radiomics features of BS.

## 1. Introduction

Prostate cancer (PCa) ranks as a major health concern due to its high incidence and its position as the fifth leading cause of cancer death among men. In 2020 alone, it was estimated that globally, 1.4 million men were diagnosed with PCa, and 375,000 men died of it [1]. The approach to PCa treatment is determined by considering the patient’s expected lifespan, the classification of PCa risk, and the anticipated outcomes of the therapy [2]. Therefore risk stratification and recurrence prediction are pivotal when diagnosing and treating PCa [2]. Currently, the D’Amico classification and the AJCC (American Joint Committee on Cancer) TNM system serve as the cornerstone for PCa risk stratification and prognosis prediction [2,3]. However, despite their widespread adoption, these classification systems still fall short of accurately predicting the varied behaviors of localized or locally advanced PCa [4]. This highlights a pressing need for a more refined risk stratification system and the development of a robust recurrence prediction model. In the last two decades, the health sector has turned to Artificial Intelligence (AI) for managing extensive datasets and optimizing patient care [5]. Machine Learning (ML), as a subset of AI, has been applied to various facets of PCa, including predicting outcomes of robotic radical prostatectomy (RP), assessing radiation therapy (RT) responses, and differentiating metastatic bone lesions associated with PCa [6,7,8,9]. Such advancements reinforce the notion that ML models, which encompass features across multiple dimensions, can potentially further predict survival outcomes and refine risk stratification. 

The AJCC TNM system relies on conventional imaging; thus, the European Association of Urology (EAU) and NCCN guideline recommend both computed tomography (CT) and bone scintigraphy (BS) as essential tools for assessing the extent of disease [2,10]. Although BS is an important tool for PCa staging, metastasis detected by BS is only 0.8% and 10% of intermediate and high-risk PCa, respectively [11]. The low detection rate of metastatic PCa and non-specific findings are the major limitations of BS [12]. The failure of BS to detect metastasis partly explains the early recurrence or biochemical recurrence (BCR) in a quarter to half of patients who were initially diagnosed with localized PCa [13,14]. Since undetected metastasis in BS is a contributing factor to early recurrence in patients initially diagnosed with, or misclassified as having, localized or locally advanced PCa [13]. Improving the detection rate of BS remains an imperative issue yet to be addressed. Radiomics, a novel method of ML, extracts data from medical images and transforms them into quantitative features using bioinformatics approaches [15,16]. Radiomics features have shown the potential to discover disease patterns or metastatic lesions that were unnoticed by the human eye [15,17]. Furthermore, radiomics features have been demonstrated its efficacy in early disease detection across various cancers [18]. The aforementioned literature provides a theoretical foundation for the role of radiomics in enhancing BS and improving cancer staging or risk stratification. However, the integration of the ML model with radiomics features of BS for prognostic prediction in PCa remains underexplored. Therefore, we focused on the patients with local or locally advanced PCa who received initial treatment and dissected those with early recurrence. This study aims to develop and compare different types of ML-trained recurrence-prediction models of local or locally advanced PCa by combining clinical features and radiomics features of BS.

## 2. Methods

### 2.1. Study Population 

This study is a retrospective analysis of patients with local or locally advanced, treatment-naïve PCa at China Medical University Hospital between 24 March 2011 and 14 November 2019. The study was approved by the local Institutional Review Board (certificate numbers: DMR99-IRB-293-(CR-11)). The inclusion criteria for the study were patients with local or locally advanced PCa as defined by board-certified urologists using the clinical, image, and pathological features based on the recommendations of NCCN or EAU guidelines [2,10]. Exclusion criteria included patients with incomplete medical records, those diagnosed with metastatic PCa, or those who were treated as if they had metastatic PCa. All patients included in this study underwent comprehensive studies including digital rectal examination, PSA testing, prostate biopsy, soft-tissue imaging (either CT or MRI) and whole-body BS as part of the routine staging procedure according to the NCCN or EAU guidelines [2,10]. 

### 2.2. Study Endpoints and Design

**Figure 1:**
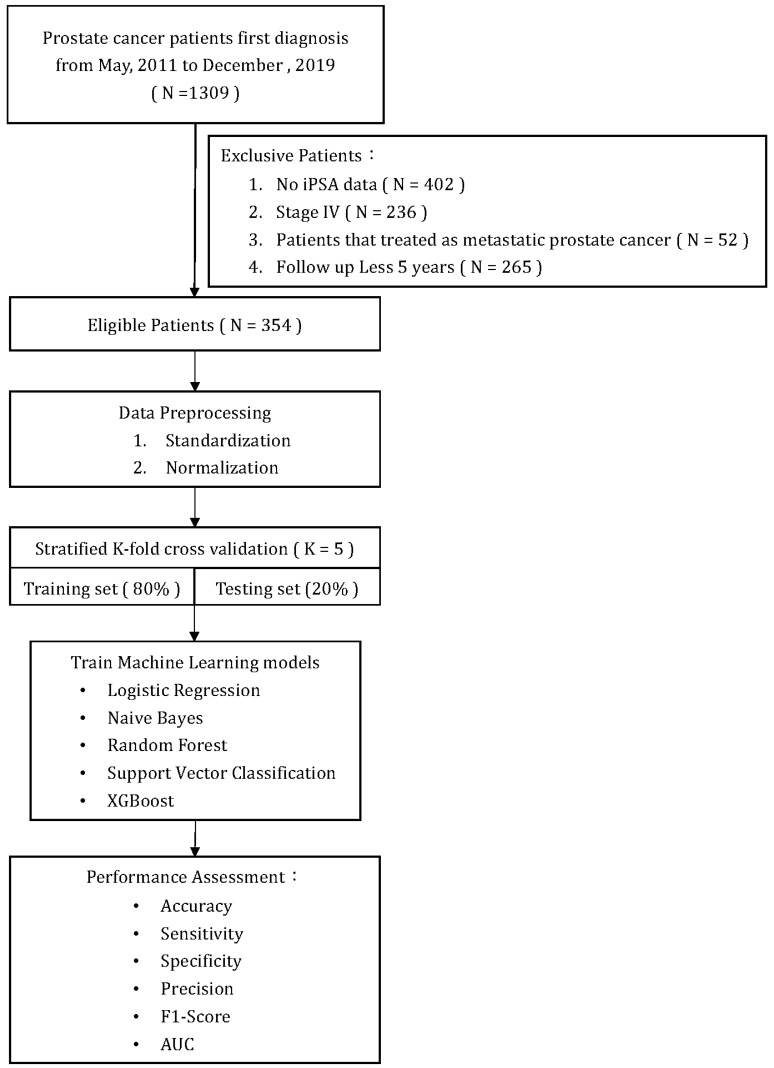
Flow chart.

Figure 1 illustrates a flowchart of patient selection and study design. A total of 354 patients who met the inclusion/exclusion criteria were included in the analysis and model training. All primary prostate lesions were confirmed by biopsy and evaluated by board-certified uropathologists. From the point of their initial diagnosis of PCa, all patients were consistently monitored for a minimum of five years or until the recurrence of the disease. Recurrence of PCa after initial treatment was defined as either the identification of rising PSA levels (BCR), radiographic progression (local recurrence, new lymph node involvement, new metastases), or death due to PCa. The definition of BCR after initial treatment was established as follows: (1) after RP: as a serum PSA ≥ 0.2 ng/mL followed by a second confirmatory level; (2) after RT, cryotherapy, or high-intensity focused ultrasound (HIFU): the RTOG-ASTRO Phoenix Consensus Conference definition as any PSA increase > 2 ng/mL higher than the PSA nadir value; (3) after androgen deprivation therapy (ADT): three consecutive rises in PSA at least one week apart resulting in two 50% increases over the nadir, and a PSA > 2 ng/mL plus castrate serum testosterone < 50 ng/dL or 1.7 nmol/L. 

### 2.3. Clinical Features

The survival outcomes were analyzed based on several clinical features including age, baseline comorbidity, PSA level at diagnosis (or initial PSA), cancer stage, Gleason score of PCa, D’Amico Risk Classification, and the type of initial treatment modality. The cancer stage was defined by the AJCC TNM staging system of PCa. The extent of PCa, including lymph node involvement and distant metastasis, was assessed using conventional imaging by board-certified uro-radiologists and nuclear medicine physicians. Baseline comorbidity was categorized based on the Charlson Comorbidity Index. Gleason grade was defined by the International Society of Urological Pathology (ISUP) Consensus Conference. The initial treatment modalities were classified as active surveillance (AS), RP, RT, HIFU, cryotherapy, and adjuvant ADT. The choice of the initial treatment modality was made by the surgeon and based on patient preference. The follow-up period was defined as the interval extending from the date of the initial PCa diagnosis to the date of either disease recurrence or the most recent clinical visit.

### 2.4. Image Acquisition

Whole-body BS with 99 mTc-labeled diphosphonates is a type of nuclear imaging test that uses a radiotracer to evaluate the distribution of active bone formation in the skeleton related to cancer bone metastasis, as well as physiological processes, particularly in PCa bone metastases. All patients underwent BS within 90 days (mean ± SD, 22.5 ± 13.9 days) after being diagnosed with PCa. Routine whole-body scans were performed 2–4 h after the intravenous administration of 20 mCi of 99 mTc-labeled MDP with a scan speed of 14–17 cm/min on either a Millennium MG, Infinia Hawkeye 4, or Discovery NM/CT 670 Pro scanner (GE Healthcare, Chicago, IL, USA). Each patient’s BS produced two images, specifically anterior and posterior images, with a resolution of 1024 × 256 pixels.

#### 2.4.1. Radiomic Analysis

The body range within a user-specified region of interest (body mask) was identified using the image value relationship between each pixel and adjacent pixels. The body mask was defined using a relative threshold of background noise, specifically, an image value greater than or equal to 2. We found that this approach maximized the probability of connection between the extracted image value and the adjacent body.

**Figure 2:**
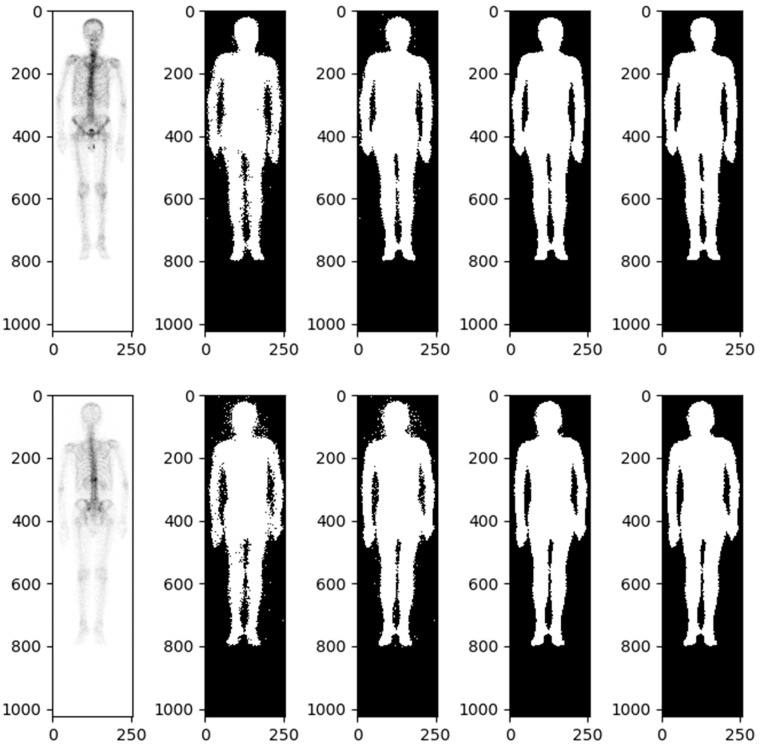
Bone scintigraphy mask for extracting radiomics features.

Details of automatic body mask or Region of Interest (ROI) creation are illustrated in Figure 2 and elaborated upon in the subsequent description. The process is started by converting the BS image into a grayscale image with pixel values ranging from 0 to 1. This is followed by dilation and erosion procedures aimed at filling minor, fragmented gaps. Noise reduction is achieved via additional rounds of erosion and dilation to eliminate extraneous white dots. At this stage, a preliminary version of the bone skeleton mask is available. Compared to other medical images, BS images are simpler. The ROI for the entire body can be extracted using basic image processing techniques rather than AI. Moreover, professional nuclear medicine physicians have also validated all these ROI images. The next step involves eliminating the smallest interconnected regions, such as superfluous catheters. The final step in the procedure is to fill any remaining blank spaces within the image, resulting in a mask designed for the extraction of radiomics features (Figure 2).

A morphological closing and morphological opening algorithm was applied to separate the body and adjacent anatomic structures if they were misclassified. The software used for feature extraction was developed by pyradiomics 3.1.0. For each BS image, 64 radiomics features, including first-order statistics and texture features, were extracted from the ROI. The BS images were classified into two groups, front and back, resulting in a total of 128 radiomics features for each patient. Furthermore, in order to calculate texture features, the values within the ROI were discretized. To describe the heterogeneity of the discretized value within the ROI, four textural matrices were calculated: the gray-level cooccurrence matrix (GLCM), the neighboring gray-level dependence matrix (NGLDM), the gray-level run-length matrix (GLRLM), and the gray-level size zone matrix (GLSZM).

#### 2.4.2. Data Imbalance and Oversampling

We incorporated the Imblearn function to the model to randomly resample the imbalanced data. The two types of labels of our data are 253 and 101, respectively. Imbalanced data occurs in the classification task. This situation is common and requires rectification. To address this, we utilized both the methods of duplicating the minority class and reducing the majority class. We employed the approach of SMOTE (Synthetic Minority Oversampling Technique) as described by Nitesh Chawla et al. in their 2002 paper, “SMOTE: Synthetic Minority Over-sampling Technique” [19]. SMOTE operates by selecting instances that are proximate in the feature space, drawing a line between these instances, and generating a new sample at a point along that line. Specifically, a random instance from the minority class is selected. Then, k of its nearest neighbors are identified. One of these neighbor is selected, and a synthetic sample is produced at a randomly chosen point between the two instances in feature space.

#### 2.4.3. K-Fold Cross-Validation

**Figure 3:**
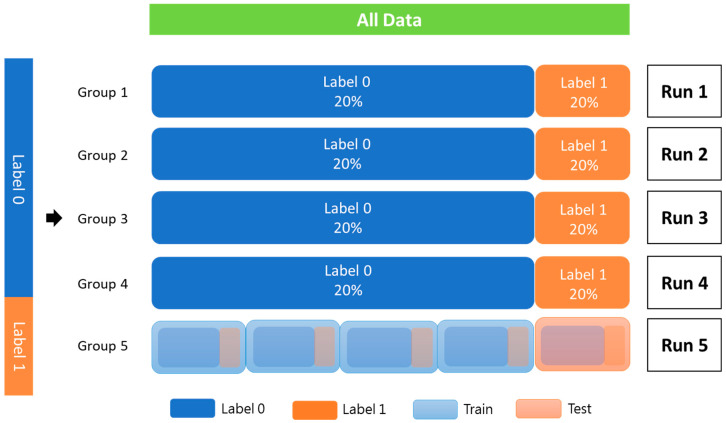
K-fold cross-validation (K = 5).

The patients were used as the training data, and StratifiedKFold from scikit-learn 1.1.3 was utilized for k-fold cross-validation to assess the robustness of the proposed model. The training cohort was divided into five groups, each with an equivalent proportion of patients with and without PCa recurrence. Each group was used only once as a test set, and the rest were combined to form a dataset for training (Figure 3). 

All data were randomly added to the index according to label 0 and label 1. Each fold should be equally distributed between label 0 and label 1, and the index cannot be repeated in another batch. The data were trained to complete one epoch. According to the typical distribution of the labeling category, a part of all the data is used as verification data. Then, the remaining data are k-folded into training and testing data. Label 0 indicates without PCa recurrence; label 1 indicates recurrence of PCa.

### 2.5. Machine Learning Model

**Figure 4:**
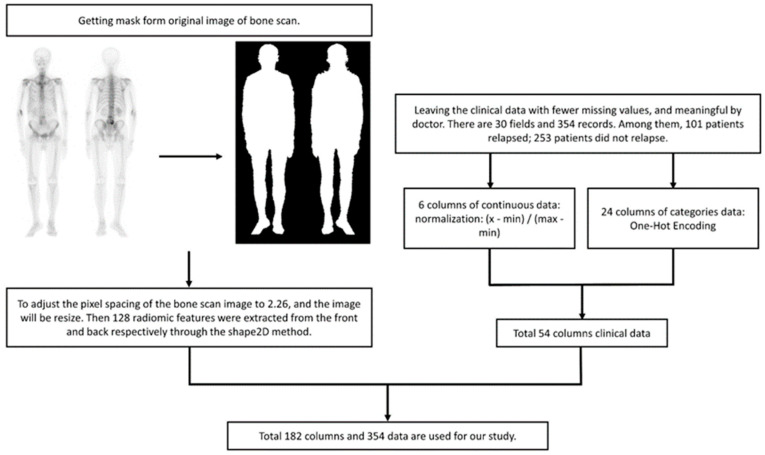
Data preprocessing flow chart. Normalization aims to compress the value between 0 and 1, and eliminate the potential influence of varying units without changing the original distribution. Radiomics features lack units and almost invariably lie between 0 and 1.

ML: For BS images, we utilized radiomic analysis to extract 128 features and combined them with continuous-type clinical data, which was transformed into a range of values from 0 to 1. The category of clinical data was converted through One-Hot Encoding and added to the training data for the model (Figure 4). The model implementation is as follows:

1. Logistic Regression: Logistic regression (LR) is a statistical model used for binary classification tasks. The LR model is an extension of linear regression, but instead of predicting continuous values, it predicts the probability of an event occurring. The predicted probability is then transformed using a logistic (sigmoid) function to ensure it lies between 0 and 1.
2. Naive Bayes: Naive Bayes (NB) is a classification algorithm based on Bayes’ theorem with the assumption of independence among the predictor variables. It assumes that the probability of a certain class, given the occurrence of other features, can be calculated using prior probabilities and conditional probabilities. In the NB classification, we are focused on calculating the probability of a class.
3. Random Forest: Random Forest (RF) is a supervised ML algorithm that is used widely in classification problems. It builds decision trees on different samples and takes their majority vote for classification or the average in the case of regression.
4. Support Vector Classification: Support Vector Classification (SVC) is based on Support Vector Machine (SVM). The goal is to create the best line or decision boundary that can segregate n-dimensional space into classes. It aims to find a hyperplane in a high-dimensional feature space that can separate the feature of different classes with the largest margin.
5. Xtreme Gradient Boosting: Xtreme Gradient Boosting (XGBoost) is based on Gradient Boosting, combined with the advantages of the bagging method of RF. Each decision tree is related to the other, and the previous errors are corrected by the decision tree generated later, with L1/L2 Regularization to avoid overfitting.

The first four models were sourced from scikit-learn 1.1.3, and the XGBoost model came from XGBoost 1.6.2.

#### Statistical Analysis

For the clinical characteristics in the 354 study patients, the numerical variables were displayed by the median and interquartile range (IQR). The categorical variables were displayed as the percentage (%). The *t*-test was employed for continuous variables, while the Chi-square (χ^2^) test was utilized for categorical variables. A *p*-value of less than 0.05 was considered statistically significant. To evaluate the effectiveness of the classifier, the area under the curve (AUC) derived from the receiver operating characteristic (ROC) curve was utilized. Performance indicators included sensitivity, specificity, precision, F1-score, and accuracy. The analysis was conducted using SPSS software (version 26; IBM, Armonk, NY, USA).

## 3. Results

### 3.1. Patient Characteristics

In the baseline characteristics, 253 patients (71.47%) were categorized into the non-recurrence group, while 101 patients (28.53%) were classified into the recurrence group (Table 1). The mean age in both groups was close to 70 and there was no significant difference in baseline comorbidities between the groups (Table 1). The recurrence group had a higher median initial PSA level (10.23 ng/dL, *p*-value < 0.052), a higher-grade group (42.57% with grade group > IV, *p*-value < 0.01), a more advanced T stage (65.35% with T ≥ 3, *p*-value < 0.01), more lymph node involvement (19.80% vs. 8.30%, *p*-value < 0.01), and a higher proportion of high-risk patients (73.27% vs. 43.48%, *p*-value < 0.01) (Table 1). Additionally, more patients underwent RP in the recurrence group compared to the non-recurrence group (80.20% vs. 55.73%, *p*-value < 0.01). 

**Figure 5:**
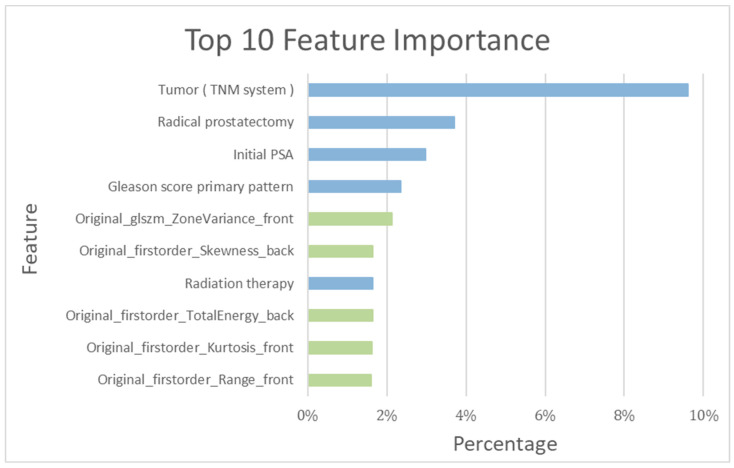
Top 10 features’ importance observed in the RF_clinical and bone scintigraphy radiomics model. Blue bar: clinical feature; green bar: bone scintigraphy radiomics feature.

Among the top ten important features determined by the training model, the following five clinical features were included: tumor stage (TNM system), RP, initial PSA, Gleason Score primary pattern, and radiotherapy (RT) (Figure 5).

### 3.2. Patient-Based Prediction 

As detailed in Table 1, a total of 354 patients in the training cohort were assessed both with and without recurrence. The recurrence-prediction model employing ML was subsequently analyzed. Among the top ten salient features highlighted by the training model, the following five radiomics features were included: Gray Level Size Zone Matrix, Skewness, Total energy, Kurtosis, and Range (as shown in Figure 5). The RF_clinical and BS radiomics model, boasting the best performance metrics with a final AUC of 0.94, was utilized for making predictions based on the BS radiomics from the 5-fold assessment of the 354 patients. 

The performance metrics of the RF_clinical and BS radiomics model are as follows: accuracy (0.85), sensitivity (0.81), specificity (0.87), precision (0.71), F1-score (0.76), and AUC (0.94). The results of other training models displayed a range of accuracies (0.52 to 0.78) and AUCs (0.52 to 0.78), as detailed in Table 2. A confusion matrix details the performance of the RF_clinical and BS radiomics classification model, as illustrated in Table 3, with a precision of 0.71 and a recall of 0.81.

**Figure 6:**
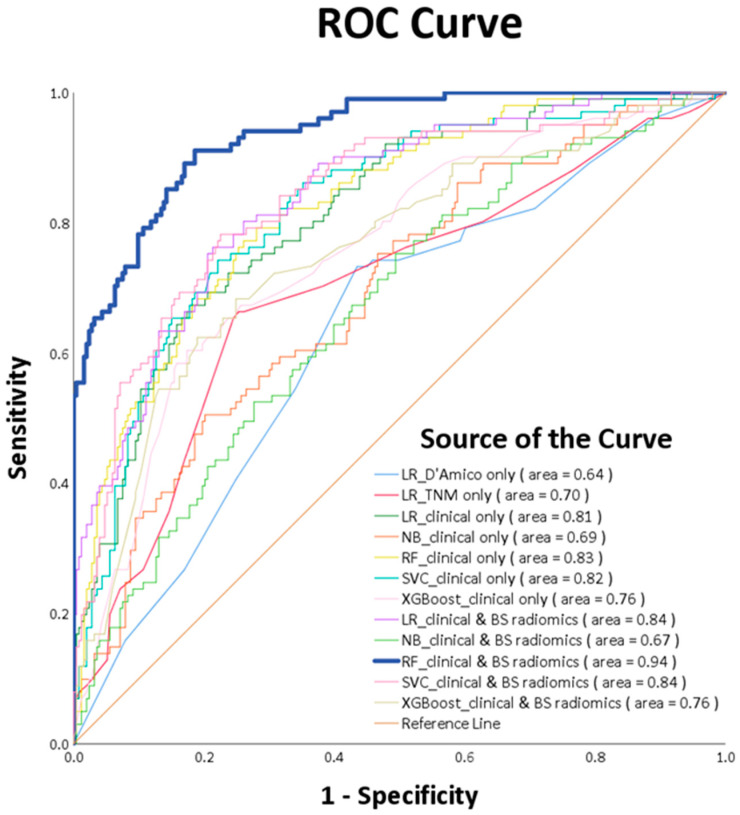
ROC curves of various machine learning models.

ROC curve analysis ascertains that the RF_clinical and BS radiomics predictions surpassed the performance of other ML models, registering a culminating AUC of 0.94 (*p*-value < 0.0001), as visualized in Figure 6. A comparative review of the receiver operating characteristic (ROC) for the RF_clinical and BS radiomics model against the other nine models is delineated in Table 4. This confirms that the RF_clinical and BS radiomics model notably outperformed the rest.

## 4. Discussion

Our study aimed to evaluate the usefulness of ML based on multiple clinical features and radiomics features of BS in differentiating PCa recurrence. In total, 354 local or locally advanced PCa patients who underwent comprehensive studies, including BS before initial treatment, were enrolled in the study retrospectively. The radiomics features of BS were extracted and calculated within a body mask frame. All images were automatically extracted using an algorithm approved by experts. The results showed that combining the clinical features and radiomics features of BS, the ML-trained model could predict the recurrence status after initial treatment. Moreover, the RF_clinical and BS radiomics model, which effectively predicts recurrence status, is the best-performing model. These findings may facilitate personalized medical care by aiding physicians in identifying patient groups with a high risk of recurrence. The sensitivity and specificity of the RF_clinical and BS radiomics model were 0.81 and 0.87, respectively. In an ideal scenario, a diagnostic test would have 100% sensitivity and specificity; however, such outcomes are rarely observed in practice. The specificity of our model surpassed its sensitivity. A diagnostic test with high specificity suggests that if the test result is positive, there’s a high probability of future recurrence. Clinically, this indicates the potential need for more intensive adjuvant treatments or more vigilant follow-up strategies post initial therapy [2,10]. Conversely, we can also differentiate patients in the low-risk recurrence group, thereby preventing the potential of overtreatment. 

Currently, the D’Amico classification and the AJCC (American Joint Committee on Cancer) TNM system are the most widely used methods for PCa risk stratification and prognosis prediction [2,3]. Zelic et al. demonstrated that a more detailed risk stratification provided better discrimination, with the MSKCC nomogram and CAPRA score performing better than the D’Amico and TNM systems [20]. This inspired the development of a more sophisticated risk stratification system, which might be achieved with the support of the ML model. In a multicenter study, ML model methods outperformed LR in predicting clinical deterioration on the ward [21]. Furthermore, it demonstrated that the RF algorithm surpassed other ML methods in accuracy [21]. Deist et al. compared various ML models, including decision trees, RF, neural networks, SVM, elastic net logistic regression, and LogitBoost. They found that the RF and elastic net logistic regression exhibited superior discriminative performance in (chemo)radiotherapy outcomes across 12 different cancer datasets [22]. Similar to previous studies, in this research, the RF outperformed all other ML models in predicting the disease progression. The RF excels in clinical data analysis, offering high accuracy by integrating multiple decision trees, each based on various sub-datasets from the original data [23]. The introduction of randomness in RF prevents overfitting, enhancing adaptability to new data amid clinical complexity. Therefore, RF can improve accuracy over single prediction models, even with smaller datasets [23]. RF is well-suited for the high-dimensional and irregular scales found in clinical data and can handle numerous features and assess their importance. Instead of selecting only the best feature, RF trains on diverse feature subsets [24]. Notably, RF is insensitive to data scale and does not require feature regularization, contributing to practicality in clinical applications. The ensemble nature of RF effectively addresses imbalanced data distribution, ensuring consistent performance across diverse sample categories. Given these attributes, it’s evident that RF is especially tailored for deciphering the intricacies of medical datasets.

Current risk stratification systems for PCa consider a restricted set of factors and employ simple grading. Consequently, they fall short in addressing the pronounced heterogeneity inherent to PCa. For instance, neither the D’Amico nor the TNM system takes into account crucial survival-related factors such as age and comorbidities, both of which were incorporated in our study [25,26]. Another drawback of the existing PCa risk stratification system is that it treats each constituent factor with equal importance, which may differ from the heterogeneity observed in PCa. Moreover, the significance weight of each factor should take into account the accuracy of each respective test. For example, Gleason scoring is based on biopsy samples that might not represent the entire prostate, and the PSA value can be affected by age and benign conditions [27,28]. In our study, the predictive value based on the D’Amico or TNM system was less powerful than the ML models. Conventional prognostic models primarily rely on linear regression, which can potentially mis-weight contributing factors. This may lead to inaccuracies in risk stratification and the prediction of PCa recurrence. ML models, by presenting a nonlinear, multidimensional perspective on the heterogeneous biology of cancer, offer solutions to these challenges and have proven their applicability across various diseases [29]. In addressing the heterogeneity of PCa, Lee C et al. verified that ML algorithms, which build multivariable models through the automatic integration of optimal attributes, outperform conventional tiered risk stratification systems [30]. Compared to deep learning, ML algorithms are more suitable for the context of this study, which involves a smaller sample size and single-label classification. In our study, combining the clinical features with the image features showed improved predictive accuracy for PCa recurrence compared to clinical features alone, similar to previous studies in head-and-neck and lung cancer [31]. Taken together, we presented an initial attempt to show that an ML-trained prediction model using multiple clinical features and radiomics features of BS can enhance the accuracy of PCa prognosis and provide individualized risk assessments tailored to specific patient characteristics.

With the aid of AI, it is possible to detect early PCa bone metastasis and uncover bone lesions in BS [32]. Through radiomics, image data are transformed into first-order features (which describe non-spatial values), second-order features (commonly referred to as “texture” features), and higher-order features [16]. Radiomics features have been found to reflect tumor biology and are significantly associated with cancer genomics, proteomics, and metabolomics, helping to capture the spatial heterogenicity of PCa [33]. The aforementioned literature provides a foundational rationale for enhancing the diagnostic power of BS through radiomics. Radiomics consists of three main parts: segmentation of the ROI, extraction of image properties, and statistical analysis/modeling of these properties [16]. Pyradiomics, which was used in our study, supports both feature extraction in 2D and 3D and can be used to calculate single values per feature for a ROI or to generate feature maps. However, the difference in the same feature in different classes is more important than the number of features. Usually, the number of extracted 2D radiomics features was less than that of 3D features because 2D features were extracted based on a single slice. Though the difference in the same feature across different classes is more important than the number of features, it has been pointed out that 2D features showed better performance by Chen Shen et al. [34]. Considering the cost of the radiomics features calculation and the cost of medical imaging, 2D features are more recommended for extracting radiomics features from images of BS. The Radiomics Quality Score (RQS) serves as a benchmarking tool for appraising radio-histology studies based on their features and the quality of reporting [35]. In this context, our study achieved an RQS score of 16 points (44.44%). This score is notably higher than the average RQS observed in previous studies (mean RQS of 23% ± 19.6%), underscoring the robustness of our research [36].

Our study utilized the recurrence of PCa as a reference, identifying five radiomics features—Gray Level Size Zone Matrix, Skewness, Total Energy, Kurtosis, and Range-as significant for PCa prognosis. These radiomics features are described as follows: Gray Level Size Zone Matrix signifies spatial relationships; Skewness represents the asymmetry of pixel intensities; Total Energy corresponds to the sum of pixel intensity; Kurtosis quantifies the degree of tailedness; and Range describes the overall spread or variability of the intensity values. The association between radiomics features and biological correlates remains an area ripe for in-depth exploration. In the realm of PCa research, there is limited research directly linking radiomics to biology [36]. However, certain radiomic features, such as Skewness, Kurtosis, Range, and GLCM, have shown associations with bone density and osteoporosis [37]. Furthermore, research indicates that CT radiomics features can differentiate between significant RANKL status, a crucial pathway in bone modeling [38]. Zhang W et al. underscores the role of the bone metastasis in the formation of secondary metastasis, as evidenced by the progression of PCa cells from primary bone metastasis, tracked using an evolving barcode system [39]. These findings hint at a potential connection between radiomics features and the bone micro-environment or turnover. The exact pathways or genetic regulations underpinning these associations warrant further investigation. The subsequent evidence underscores the clinical significance of the radiomics features highlighted in our research. Spohn SKB et al. showed that an MRI-based radiomics feature (Gray Level Size Zone Matrix) could predict the BCR following RP, with a reported accuracy of 0.78 [40]. Skewness obtained from 18F-fluoride and 18F-FDG positron emission tomography, was independently correlated with the progression-free survival of breast cancer [41]. Moreover, CT-based radiomics features including total Energy and Kurtosis, predicted the BCR of PCa following RT [40]. Consistent with the previous literature, we offer a unique perspective suggesting that these five radiomics features represent the heterogeneous texture, which may assist in identifying micrometastatic or undetected bone lesions and predicting the recurrence of PCa.

Briefly speaking, our study obtained multiple clinical features and body images, and then fed them into the radiomics and ML to predict whether PCa would recur, followed by an assessment of accuracy. To our knowledge, this study is the first to assess the prognostic value of ML for clinical features combined with the automated classification of BS. However, our study is not without limitations as follows. Firstly, our study is primarily limited by the fact that the research population consisted solely of Taiwanese individuals. Consequently, the results may primarily be generalized to the Asian population. The applicability of these findings to other ethnicities is inherently constrained, primarily due to the well-established variations in PCa across different racial groups [42]. Second, the lack of later-stage treatment and toxicity profile resulted in an incomplete view of the entire therapeutic course, but this is beyond the scope of our study. Third, our study had a short follow-up duration, leading to an imbalance between the recurrence and non-recurrence groups. This could affect model training, but we implemented SMOTE methods to address this limitation [19]. While we do not have mature overall survival data, previous studies have indicated that recurrence-free survival is a pertinent indicator of overall survival [43]. Lastly, the use of training and testing sets does not replace full external validation. To address this issue, we tested our models with a five-fold cross-validation scheme, which showed high stability and is widely used in the field of computer-aided detection [44]. In future endeavors, we eagerly anticipate collaborations with various medical institutions across different nations to address issues related to generalizability, racial differences, and external independent validation. Furthermore, we plan to extend the follow-up period to capture complete overall survival outcomes and toxicity profiles for each treatment, thereby offering a more holistic perspective on long-term PCa treatment.

## 5. Conclusions

Our study showed that combining significant clinical features and radiomics features of BS enhances the prediction of local or locally advanced PCa recurrence after initial treatment. These results not only highlight the added value of ML techniques in risk stratification for PCa but also serve as a proof of concept that ML models, combined with radiomics features from BS, can be effectively applied to PCa. We believe the ML model with radiomics features of BS will aid in identifying patients at high risk of recurrence. Nevertheless, larger-scale multicenter prospective studies are essential in the future to validate the application of ML and radiomics features for recurrence prediction, and to confirm their predictive capacity for overall survival and toxicity profiles across various stages of PCa.

## Figures and Tables

**Table 1:** Clinical characteristics in 354 study patients.

	PCa Population		
	No Recurrence	Recurrence	
Population	*N* = 253 (71.47%)	*N* = 101 (28.53%)	*p* Value
^a^ **Diagnosis Age** *	70 (64–75)	69 (64–74)	0.38
^b^ **Comorbidity**			
Cancer (exclude Leukemia/Lymphoma)	45 (17.79%)	15 (14.85%)	0.51
Lymphoma	1 (0.40%)	0 (0.00%)	0.53
Metastatic solid tumor	4 (1.58%)	1 (0.99%)	0.67
Chronic pulmonary disease	41 (16.21%)	10 (9.90%)	0.13
Peripheral vascular disease	3 (1.19%)	0 (0.00%)	0.27
Prior myocardial infarction	5 (1.98%)	3 (2.97%)	0.57
Congestive heart failure	7 (2.77%)	2 (1.98%)	0.67
Cerebrovascular disease	33 (13.04%)	13 (12.87%)	0.97
Dementia	16 (6.32%)	6 (5.94%)	0.89
Peptic ulcer disease	57 (22.53%)	16 (15.84%)	0.16
Diabetes	39 (15.42%)	19 (18.81%)	0.44
Diabetes with chronic complications	19 (7.51%)	7 (6.93%)	0.85
Mild liver disease	15 (5.93%)	6 (5.94%)	1.00
Moderate or severe liver disease	0 (0.00%)	1 (0.99%)	0.11
Moderate-to-severe renal disease	44 (17.39%)	14 (13.86%)	0.42
Rheumatologic disease	2 (0.79%)	0 (0.00%)	0.37
^a^ **Initial PSA** *	10.56 (7.02–19.77)	10.23 (9.34–29.42)	0.05
^b^ Value ≤ 10 ng/mL	116 (45.85%)	30 (29.70%)	<0.01
^b^ Value > 10 ng/mL and Value ≤ 20 ng/mL	76 (30.04%)	32 (31.68%)	0.76
^b^ Value > 20 ng/mL	61 (24.11%)	39 (38.61%)	<0.01
^b^ **Gleason score primary pattern** *	4 (3–4)	4 (4–4)	<0.01
1	0 (0.00%)	0 (0.00%)	
2	6 (2.37%)	0 (0.00%)	
3	90 (35.57%)	19 (18.63%)	
4	146 (57.71%)	75 (74.26%)	
5	11 (4.35%)	7 (6.93%)	
^b^ **Gleason score secondary pattern** *	4 (3–4)	4 (3–5)	<0.01
1	0 (0.00%)	0 (0.00%)	
2	6 (2.37%)	1 (0.99%)	
3	90 (35.57%)	43 (17.00%)	
4	146 (57.71%)	28 (27.72%)	
5	11 (4.35%)	29 (28.71%)	
^b^ **Gleason Score_Grade Group**			<0.01
I	27 (10.67%)	4 (3.96%)	
II	68 (26.88%)	14 (13.86%)	
III	96 (37.94%)	40 (39.60%)	
IV	19 (7.51%)	9 (8.91%)	
V	43 (17.00%)	34 (33.66%)	
^b^ **T**			<0.01
T1	30 (11.86%)	4 (3.96%)	
T2	161 (63.64%)	31 (30.69%)	
T3	60 (23.72%)	58 (57.43%)	
T4	2 (0.79%)	8 (7.92%)	
^b^ **N**			<0.01
N0	232 (91.70%)	81 (80.20%)	
N1	21 (8.30%)	20 (19.80%)	
^b^ **Risk classification**			<0.01
Low risk	18 (7.11%)	3 (2.97%)	
Intermediate risk	125 (49.41%)	24 (23.76%)	
High risk	110 (43.48%)	74 (73.27%)	
^b^ **Bone scintigraphy Result**			0.41
No definite bone metastasis	241 (95.26%)	94 (93.07%)	
Equivocal findings	12 (4.74%)	7 (6.93%)	
^b^ **Cancer Treatment**			
RP	141 (55.73%)	81 (80.20%)	<0.01
RP and RT	10 (3.95%)	0 (0.00%)	0.04
RP and ADT	5 (1.98%)	3 (2.97%)	0.57
RP and ADT and RT	9 (3.56%)	3 (2.97%)	0.78
RT	56 (22.13%)	7 (6.93%)	<0.01
RT and ADT	19 (7.51%)	4 (3.96%)	0.22
HIFU	1 (0.40%)	0 (0.00%)	0.53
Cryotherapy	3 (1.19%)	1 (0.99%)	0.88
First-generation antiandrogen	2 (0.79%)	0 (0.00%)	0.37
AS	7 (2.77%)	2 (1.98%)	0.67
^a^ **Cancer Follow-up period (Years)** *	6.4 (5.51–7.54)	2.08 (1.24–3.43)	<0.01

* Values are denoted as the median (IQR); ^a^ independent sample *t*-test; ^b^ chi-square (χ^2^) test. The Gleason Score is as follows: the first number represents the cells that make up the most significant tumor area; the second number, the cells from the next largest area. The Grade group has five categories based on pathological characteristics. T denotes tumor; N stands for nodes; RP signifies radical prostatectomy; RT represents radiation therapy; ADT indicates androgen deprivation therapy; HIFU means high-intensity focused ultrasound; and AS stands for active surveillance.

**Table 2:** Comparison of the performance of different prediction models (Cross-validated data).

	k-Fold Test Result (K = 5)					
Machine Learning Model	Accuracy	Sensitivity	Specificity	Precision	F1-Score	AUC
LR_D’Amico only	0.61 (0.61–0.61)	0.73 (0.67–0.79)	0.56 (0.52–0.59)	0.40 (0.32–0.44)	0.52 (0.45–0.57)	0.64 (0.62–0.68)
LR_TNM only	0.73 (0.72–0.73)	0.65 (0.53–0.81)	0.75 (0.68–0.81)	0.52 (0.50–0.57)	0.58 (0.51–0.62)	0.70 (0.69–0.74)
LR_clinical only	0.75 (0.70–0.81)	0.71 (0.57–0.79)	0.76 (0.74–0.82)	0.55 (0.48–0.63)	0.62 (0.52–0.70)	0.81 (0.74–0.85)
NB_clinical only	0.52 (0.49–0.54)	0.87 (0.75–1.00)	0.38 (0.29–0.41)	0.36 (0.33–0.39)	0.51 (0.46–0.56)	0.69 (0.61–0.81)
RF_clinical only	0.76 (0.70–0.87)	0.71 (0.58–0.90)	0.78 (0.71–0.86)	0.56 (0.48–0.71)	0.63 (0.56–0.79)	0.83 (0.76–0.94)
SVC_clinical only	0.77 (0.74–0.81)	0.71 (0.63–0.82)	0.80 (0.74–0.88)	0.59 (0.54–0.67)	0.64 (0.60–0.70)	0.82 (0.80–0.86)
XGBoost_clinical only	0.73 (0.70–0.77)	0.64 (0.53–0.73)	0.77 (0.71–0.83)	0.53 (0.46–0.59)	0.58 (0.53–0.62)	0.76 (0.74–0.79)
LR_clinical and BS radiomics	0.78 (0.73–0.81)	0.76 (0.57–0.90)	0.79 (0.75–0.82)	0.59 (0.52–0.64)	0.66 (0.55–0.71)	0.84 (0.76–0.89)
NB_clinical and BS radiomics	0.51 (0.44–0.54)	0.82 (0.70–1.00)	0.38 (0.29–0.47)	0.35 (0.30–0.39)	0.49 (0.42–0.56)	0.67 (0.60–0.81)
RF_clinical and BS radiomics	0.85 (0.76–0.99)	0.81 (0.60–1.00)	0.87 (0.73–0.98)	0.71 (0.54–0.96)	0.76 (0.60–0.98)	0.94 (0.83–1.00)
SVC_clinical and BS radiomics	0.77 (0.74–0.80)	0.71 (0.62–0.79)	0.80 (0.77–0.82)	0.59 (0.54–0.61)	0.64 (0.58–0.68)	0.84 (0.75–0.90)
XGBoost_clinical and BS radiomics	0.73 (0.70–0.76)	0.65 (0.53–0.73)	0.76 (0.71–0.84)	0.52 (0.46–0.56)	0.58 (0.54–0.62)	0.76 (0.75–0.80)

**Table 3:** Confusion matrix for the RF_clinical and BS radiomics prediction.

		Predicted	
		No	Yes
True	No	219	34
	Yes	19	82

**Table 4:** Comparison of receiver operating characteristic (ROC) between the best model and other models.

The Best Model	Other Model	*p*-Value
RF_clinical and BS radiomics	LR_D’Amico only	<0.0001
	LR_TNM only	<0.0001
	LR_clinical only	<0.0001
	NB_clinica only	<0.0001
	RF_clinical only	<0.0001
	SVC_clinical only	<0.0001
	XGBoost_clinical only	<0.0001
	LR_clinical and BS radiomics	0.0003
	NB_clinical and BS radiomics	<0.0001
	SVC_clinical and BS radiomics	0.0003
	XGBoost_clinical and BS radiomics	<0.0001

## Data Availability

Not applicable.
